# Similar calcifications of the brain on computed tomography, but different etiologies

**DOI:** 10.4103/0972-2327.53088

**Published:** 2009

**Authors:** Bindu Menon, C. V. Harinarayan

**Affiliations:** Department of Neurology, Sri Venkateswara Institute of Medical Sciences, Tirupati - 517 507, Andhra Pradesh, India.; 1 Department of Endocrinology and Metabolism, Sri Venkateswara Institute of Medical Sciences, Tirupati - 517 507, Andhra Pradesh, India.

Two patients with different diagnoses had near similar intracranial calcification on computed tomography (CT). In this article we present these cases.

## Case 1

**Figure 1:**
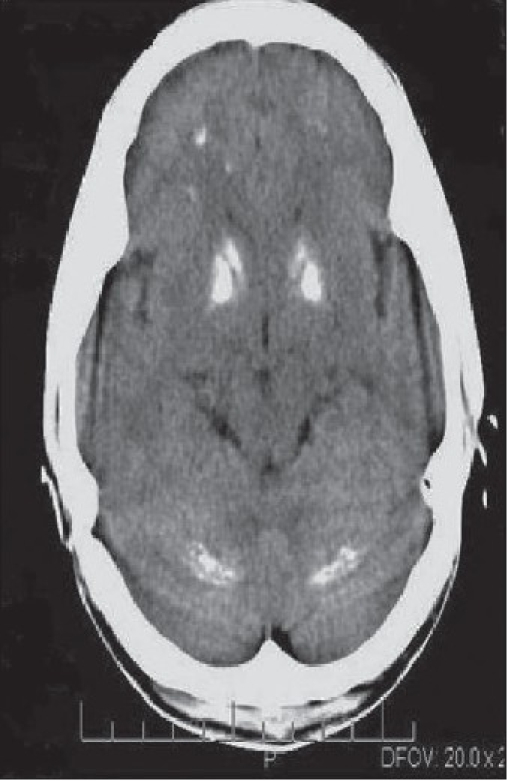
Plain CT head shows symmetrical dense calcification of the anterior lentiform nucleus, anterior limb of the internal capsule, the dentate nucleus of cerebellum, and the gray matter–white matter interface of the frontal lobe on both sides

A 16-year-old female presented with a first episode of generalized tonic–clonic seizure. On detailed enquiry, she gave history of cramps and chronic headache. There was no past or family history of epilepsy. General examination revealed grade 4 Trousseau sign and grade 1 Chvostek sign, which was suggestive of hypocalcemia. There was no focal neurological deficit. Biochemical and hormone investigations revealed hypocalcemia (4.5 mg/dl) with low intact parathormone (PTH) level (< 6.4 pg/ml), favoring a diagnosis of idiopathic hypoparathyroidism. CT head showed bilateral basal ganglia and cerebellar calcification [Figure 1]. The patient was started on daily supplementation of 0.5 μg of calcitriol and 1000 mg of elemental calcium (calcium carbonate). She has been asymptomatic for the last 2 years.

## Case 2

**Figure 2:**
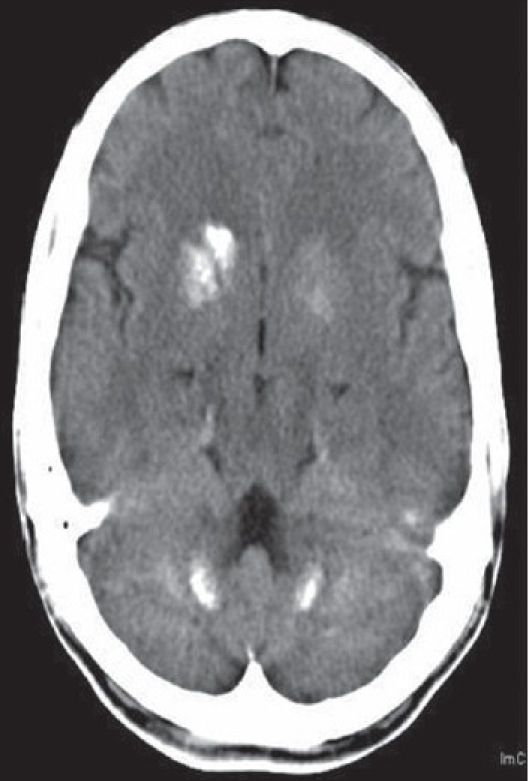
Plain CT head shows bilateral calcification in the anterior lentiform nucleus, caudate nucleus, and dentate nucleus of cerebellum

A 25-year-old male presented with episodes of complex partial seizures since 3 years. Past and family history was insignificant. His general and neurological examination was normal. Routine biochemistry revealed normocalcemia (10.1 mg/dl) and normal PTH (28 pg/ml). Plain CT head showed bilateral basal ganglia and cerebellar calcifications [Figure 2]. The patient was diagnosed to have Fahr disease[1] and was started on oxcarbamazepine 600 mg/day. This patient has been seizure-free for the last 1 year.

## Discussion

In this modern age, imaging is gaining priority over clinical examination. Indeed, neuroimaging does usually help the clinician in narrowing down the diagnosis. Intracranial calcification is occasionally an idiopathic feature and therefore detailed biochemical and hormonal evaluation is not carried out unless there is a high index of suspicion. The causes of basal ganglia calcification are many: It may be idiopathic or due to old age, hypoparathyroidism / pseudohypoparathyroidism, birth anoxia, radiation, lead and carbon monoxide poisoning, or infections such as toxoplasmosis or cytomegalovirus. Differentiating between these various possibilities solely on the basis of radiologic findings may sometimes be difficult; however, there may be certain minor differences. In old age, birth anoxia, and toxin-induced injuries, calcifications are generally confined to the globus pallidus. The calcification seen in infectious diseases is usually asymmetric and not restricted to the basal ganglia. A thorough neurological history regarding antenatal/ birth history and toxin exposure will assist in formulating the neurological diagnosis. The calcifications in Fahr disease are not distinguishable from those secondary to hypoparathyroidism. In our patients, a detailed history and clinical examination prompted us to do the biochemical/hormonal investigations and thus helped us in diagnosing these treatable cases of epilepsy.
